# “Why Are You Happy if Your Dad Died?”: The Social Experiences of Parentally Bereaved Children in Elementary and Middle Schools

**DOI:** 10.3390/children13010155

**Published:** 2026-01-22

**Authors:** Yael Boutton-Laor, Yulia Muchnik-Rozanov, Rivi Frei-Landau

**Affiliations:** 1 Achva Academic College, Arugot 79800, Israel; 2 Tel Aviv Sourasky Medical Center, Dana-Dwek Children’s Hospital, Tel Aviv 64239, Israel; 3 Department of Psychology, Ariel University, Ariel 40700, Israel

**Keywords:** childhood bereavement, orphanhood, social support, peer group, educational support

## Abstract

**Background**: Parental loss in childhood is a significant developmental risk factor, underscoring the need for evidence-based knowledge to guide support. Although social responses play a central role in children’s adjustment to loss, little is known about how parentally bereaved children in Israel experience social support in school. **Methods**: This qualitative study examined how parentally bereaved children in elementary and middle school experience social responses in the school context. Thirty-six participants were interviewed: 20 children who participated in dyadic interviews with their 16 surviving parents. Linguistic analysis, combined with Grounded Theory, was used to analyze the data. **Results**: Peer support was found to lie on a continuum ranging from support, through an unintentional lack of support, to deliberate nonsupport (teasing). These patterns shaped children’s experiences of returning to school, their sense of belonging, and their ability to share their grief. **Conclusions**: The findings, discussed in light of the Dual Process Model of Coping with Bereavement, highlight the crucial role of peers in children’s adaptation to parental loss. Developing evidence-based knowledge in this area may inform policy change and tailored school-based training to promote optimal support for parentally bereaved children.

## 1. Literature Review

### 1.1. Childhood Bereavement

The Iron Swords War led to a marked increase in the number of children in Israel who have lost a parent. Since the outbreak of the war, 1053 new orphans have been recognized by the National Insurance Institute and security services, of whom 293 are under the age of 18 [1]. Childhood bereavement is a complex experience with profound implications for children’s development [2]. Research has shown that childhood bereavement has psychological, social, health-related, and even life-threatening consequences [3]. Furthermore, the loss of a parent in childhood has been associated with lower levels of life satisfaction, even a decade after the loss [4]. Despite all this, children remain at risk of receiving limited, if any, support following the loss [5,6,7].

### 1.2. Theoretical Models of Childhood Bereavement

Bowlby’s attachment theory [8] addresses the biologically based need of children for proximity to an attachment figure, namely the caregiving figure. His work examined the observable responses following the loss of an attachment figure [9]. According to this perspective, separation from a parent is expressed in a pattern that can be divided into three stages: protest, despair, and emotional detachment from the mother figure [8]. In the protest stage, the child experiences anxiety and a strong desire to be reunited with the mother, which may be manifested in intense crying and searching for her gaze or voice. At times, children may cling to an alternative caregiving figure. The despair stage, characterized by hopelessness, is marked by quieter and more monotonous crying, an absence of demands directed toward others, and reduced arousal. In the third stage, detachment, the child ceases to reject alternative caregivers and may even accept their offers of help and play.

From an attachment-theoretical perspective, the child’s close environment plays a vital role in facilitating adaptive coping with childhood loss. Actions such as talking about the situation, open sharing and communication that allow for questions and emotional expression, involvement in rituals, and the establishment of a new, secure relationship support the child in this process [10]. In line with this, Kubler-Ross argued that children are capable of experiencing grief and highlighted the importance of including them in open conversations about the loss [11]. Indeed, children report that one of the most meaningful forms of support for them is the expression of comfort, primarily by adults but also by peers. In addition, they wish to receive help in preserving the memory of the deceased through storytelling [12].

A contemporary theoretical framework is the Dual Process Model of Coping with Bereavement (DPM) that conceptualizes grieving as a dynamic process in which bereaved individuals oscillate between two complementary orientations: loss-oriented coping, focused on confronting the pain of the loss and processing grief-related emotions, and restoration-oriented coping, focused on adapting to life changes, assuming new roles, and engaging in everyday tasks and distractions [13]. Rather than viewing healthy adjustment as linear “resolution” of grief, the DPM emphasizes flexible movement back and forth between these orientations, whereby periods of engagement with the loss alternate with periods of respite in which attention turns to restoration and ongoing life demands [13,14]. This oscillation is considered adaptive, as it allows for both emotional processing and functional adjustment over time, while also acknowledging that stressful triggers in the environment (such as reminders, anniversaries, or social interactions) can temporarily shift the bereaved person back into a loss-oriented focus [14].

Another relevant theory is the Two-Track Model of Bereavement, developed by Rubin [15,16], which offers a theoretical, clinical, and research-oriented conceptualization of adaptation to loss along two axes. Track I addresses bio-psycho-social functioning, whereas Track II refers to a more covert dimension that includes the relationship with the deceased [16]. It has been argued that among bereaved children, the functional track may also encompass school functioning, both social and academic, as well as the return to routine [17]. In addition, it has been suggested that attention should be given to relationships with other significant figures such as parents and teachers, and possibly even peers. This is consistent with Bronfenbrenner’s ecological framework [18], which emphasizes the integrated ecological impact of the various systems surrounding the child throughout development.

### 1.3. Social Support in the Context of Bereavement

In general, perceived social support in times of stress has been found to be positively associated with psychological well-being and subjective happiness, and negatively associated with depression, anxiety, and stress [19,20]. Specifically, in cases of bereavement under violent (e.g., suicide or homicide) or sudden circumstances (e.g., road accidents or natural disasters), social support has been linked to a reduced risk of PTSD [21], as well as to higher levels of posttraumatic growth [22]. Social support has also been positively associated with psychological well-being among individuals who have lost a loved one to suicide [21]. Studies by Aoun et al. classified sources of post-loss support into three categories: informal support, community support, and professional support [23]. It has been argued that bereaved individuals rely more heavily on informal support provided by family and friends than on professional support. Emotional support in the context of traumatic bereavement may be expressed through being physically present with the bereaved, listening attentively while focusing on their needs, and refraining from imposing time limits on their grief [24]. The literature further indicates that social support is particularly salient for children and adolescents [25]. While children perceive adult support as essential for coping with bereavement, they also report that support from peers is indispensable [12].

### 1.4. Social Adjustment of Bereaved Children

Studies have shown that adolescents who have experienced the loss of a loved one report that their bereaved status distinguishes them from their peers in two main ways [26,27]. First, they feel that they are the only ones in their social environment who have lost someone close. Second, they report that others treat them differently compared to how they were treated prior to the loss. As a result, they experience feelings of loneliness, shame, and fear of judgment, which in turn lead to a reduction in their circle of non-bereaved friends following the loss. Lytje [28], who studied bereaved children aged 9–17, found that the fear of feeling different gives rise to an internal struggle between the wish to confide in friends—hoping for understanding, and the fear that such disclosure will further accentuate the distinction between them and their peers. Moreover, studies have found that bereaved children sometimes also experience negative social interactions related to their loss, including teasing, harassment, and deliberate harm by peers around the topic of bereavement. For example, parental bereavement has been associated with a higher risk of being bullied among adolescents [29].

In a study of 35 children aged 5–15 who had lost a parent, 20% reported being subjected to such taunting [30]. The forms of teasing included negative comparisons between the bereaved child’s family and that of the aggressor (e.g., “I have a dad and you don’t”), disparaging remarks about the deceased (e.g., “I didn’t like your dad [who died]”), social avoidance of the bereaved child, and mocking the child’s sorrow or coping process. These provocations appeared to leave some bereaved children in shock, making it difficult for them to respond openly. Some reacted with aggression, and in only one case did peers present in the situation intervene to defend the child.

### 1.5. The Role of the Educational Environment in Supporting the Bereaved Child

Children spend most of their time at school, which is often perceived by them as a “second family” [31]. Lytje [32] followed the school return of bereaved children aged 9–17 who had lost a parent. Typically, the first day back at school was characterized by both anticipation about returning to a relatively normal environment and the realization that bereavement also changes the social reality at school. First, the way classmates received the bereaved child differed depending on whether the school staff had prepared them in advance. While some participants reported being welcomed warmly and openly, others described hesitation and confusion among their peers regarding how to respond appropriately, which at times led to experiences of loneliness. Bereaved children felt different both when they were asked about the loss and when the loss was not mentioned at all. According to the participants, this stemmed from the fact that classmates and teachers did not know how to talk about the subject. Upon returning to school, children who had lost a parent were often forced to choose between two social alternatives: (a) telling their classmates what had happened, thereby risking discomfort; or (b) pretending that nothing had changed. Students invested considerable energy in trying to fit in and avoid saddening their peers. However, those who maintained this façade over time often reported feeling different and sensing that classmates were avoiding them [28].

In sum, social responses constitute a significant factor in the bereavement process among children. Yet, whereas substantial research has examined childhood bereavement in the school context in other countries [33], research in Israel remains scarce. Developing relevant knowledge is particularly crucial in the Israeli context following the events of 7 October. Accordingly, the research question was formulated as follows: How do parentally bereaved children in elementary and middle school experience the social support toward them in the school setting?

## 2. Methodology

### 2.1. Study Design

Specifically, the study employed a grounded theory design, a qualitative methodology aimed at generating theory that is inductively derived from, and closely anchored in, the data collected in the field [34]. Grounded theory is characterized by iterative cycles of data collection and analysis, in which emerging concepts guide subsequent sampling and questioning (“theoretical sampling”), and by the constant comparative method, whereby incidents are systematically compared within and across cases to refine categories and their properties. Rather than testing a priori hypotheses, grounded theory seeks to construct a mid-range, empirically grounded explanatory framework that captures participants’ perspectives, social processes, and meanings in context. In the present study, this approach enabled a nuanced conceptualization of children’s social experiences following parental loss, as these experiences were allowed to shape the analytic categories and the emerging theoretical model, rather than being forced into predefined constructs [34].

### 2.2. Participants and Sampling

The study sample consisted of 20 children and adolescents aged 7.5–16 (with one participant aged 18 at the time of the interview. This participant was still enrolled and was about to complete high school at the time of the interview), as well as 16 parents (the surviving parent), including 12 mothers and 4 fathers aged 40–58. Overall, 36 participants. Inclusion criteria were at least one year since the loss—due to ethical reasons as well as methodological reasons (we assume that at least a year is needed in order to reflect about the social experience). All families took part in activities offered by the Sunflowers Non-profit Organization, which supports children who have lost a parent. Out of the children, four participants fully completed the Sunflowers program (i.e., initially took part in the Sunflower’s peer-support program and later served as peer mentors within the Sunflowers program).

Among the child participants, birth-order positions included firstborns (8), middle children (3), youngest children (7), and only children (2). Parents’ occupations are reported using broader occupational categories rather than specific job titles, in order to reduce identifiability while preserving the descriptive value of the sample. Accordingly, parents’ occupations were grouped into four categories: Healthcare & therapy (n = 6, 30%), Education (n = 3, 15%), Business/Management/Marketing (n = 7, 35%), and Tech/Engineering/Design (n = 4, 20%). Causes of parental death included illness (19) and suicide (1). A further distinction was made between deaths following a prolonged period of illness (8) and sudden deaths (11). Within the sample, there were two sibling pairs (one pair of twins and one pair of sisters). However, interviews were conducted separately with each child and the parent to protect confidentiality and minimize cross-participant influence. Detailed demographic characteristics are presented in Table 1.

### 2.3. Procedure and Ethics

The study was approved by Achva Academic College’s ethics committee (Approval No. 2024-234). Participants were recruited in collaboration with the Sunflowers organization using purposeful sampling. This method enables the selection of participants who are part of the target population and from whom it is possible to learn about the phenomenon [35]. All participants signed an informed consent form and were informed that they could withdraw from the study at any time. The legal guardian also signed a consent form for their child’s participation. At the end of each interview, we inquired about the well-being of the parent and child and offered them the option to contact us should any need arise. In reporting the findings, identifying details were altered to protect confidentiality, and pseudonyms were used to preserve participants’ privacy.

### 2.4. Data Collection

For ethical reasons, dyadic parent–child interviews were conducted. Depending on participants’ preferences, interviews took place either in their homes or via Zoom. All interviews were audio-recorded, with participants’ permission, to allow for subsequent transcription of the conversations. Each interview lasted between 40 and 120 min.

A two-stage interview procedure was used, a method commonly employed in bereavement research [36,37]. In the first stage, an open-ended question was presented: “I would like to understand the experience of children who have lost a parent at school. I would be happy if you could share your experiences with me. You can tell me anything that seems relevant to you.” This open invitation enabled participants to share what they perceived as most relevant. In the second stage, several follow-up questions were asked, such as: “Tell me about the way your friends treated you around the time of the loss, then and now,” and “How did you experience returning to school after the shiva (seven-day mourning period)? How did you experience your classmates’ reactions? Those of your grade-level peers?”. Participants were also given the option to be interviewed separately (the parent without the child present, or vice versa), if they so wished (upon parents’ approval). Practically, most of the children and parents preferred a dyadic interview.

### 2.5. Data Analysis

Multilevel analysis integrated grounded theory analysis (conducted by Author1 and Author3) with linguistic analysis (conducted by Author2 using AntConc software). Combining these methods allowed for triangulation, thus adding to the study’s trustworthiness [38].

*Grounded Theory analysis*: Data analysis was conducted according to the principles of Grounded Theory, using a three-stage coding process [34]. First, open coding was performed, during which segments of text were broken down into meaning units and labeled with initial codes that captured different aspects of the social experience at school. In the next stage, axial coding was applied, whereby the codes were organized into categories based on shared patterns and relationships, core axes were identified, and the dynamics between them were examined. Finally, selective coding was carried out, through which an integrative core category was developed to describe the social experience of bereaved children in the school setting, and a theoretical framework was constructed to explain the patterns of social experience of orphaned children with their classmates and the role of the educational staff. Consistent with the grounded theory approach, early interviews informed provisional coding and sensitizing concepts (e.g., peers as a central social context), while later interviews were guided by constant comparison and theoretical sampling to elaborate and refine categories (e.g., differentiating between unintentional lack of support and hostile peer responses). Accordingly, the interview guide was flexibly adjusted as analysis progressed. Throughout all stages of the analysis, the constant comparison method was employed, involving ongoing comparison between cases, codes, and categories, and repeated returns to the data in order to examine, refine, and elaborate the emerging theoretical model. During this process, we actively searched for instances that contradicted emerging categories, discussed these cases within the research team, and used them to refine category boundaries and strengthen the credibility of our interpretations.

*Linguistic analysis*: The major analytical approach of the present linguistic analysis of semantic fields. The concept of lexical or semantic fields, used by Lehrer [39,40], refers to sets of semantically related lexemes that denote a given conceptual domain and bear systematic relations to one another. In the present study, semantic field analysis was used to identify the perceptions of four program graduates children regarding the teacher’s role in their lives (These participants initially took part in the Sunflower’s peer-support program and later served as peer mentors within the Sunflowers program. They were selected because they were older than the other child participants and had more extensive experience with the focal phenomenon under study). An additional theoretical approach was Systemic Functional Linguistics (SFL), which offers a framework for examining how specific lexical choices convey emotional meaning [41]. In particular, the presence of adverbial intensifiers in descriptions of emotionally significant experiences can reflect different degrees of emotional intensity. The analysis was conducted in two phases. In the first stage, AntConc was used to identify all collocations with the lexemes *teacher* and *teachers* using the “Collocate” tool. The nature of the collocations regarding their semantic fields was analyzed in the four transcripts. In the second stage, based on the SFL approach, we identified utterances containing the lexemes teacher or teachers that were marked by adverbial intensifiers, such as very, really, (not) really, etc. Specifically, a corpus-based analysis was conducted using AntConc (version 4.2.4). The corpus consisted of transcribed interview data collected from the participants, with a total corpus size of 4356 tokens. Before analysis, the transcripts were minimally preprocessed: all files were converted to plain text, and orthographic normalization was applied where necessary. The Collocation Analysis was carried out using the Collocate tool in AntConc. A symmetrical collocation window of five words to the left and five words to the right (5L–5R) was applied. Function words (e.g., prepositions, auxiliary and modal verbs, and pronouns) were excluded from the analysis to ensure that only semantically informative lexical items were retained. The strongest collocates associated with the target lexeme (teacher) were further interpreted qualitatively and organized into semantic fields based on shared meaning relations and discourse function, following an inductive, data-driven procedure. The collocational strength values of the items included in the semantic field analysis ranged from 9.2 to 13.4, indicating relatively strong lexical associations within the corpus. Collocation strength was calculated using Mutual Information (MI) as implemented in AntConc. MI was selected because it highlights the strength of association between co-occurring lexical items beyond chance, making it appropriate for identifying salient meaning-based collocations in a relatively small, specialized corpus. The MI values of the items included in the semantic field analysis ranged from 9.2 to 13.4, indicating very strong lexical associations between the target lexeme (*teacher*) and its collocates within the corpus. Table 2 presents the semantic field analysis.

### 2.6. Trustworthiness

Qualitative research does not seek to present absolute truths but rather to achieve “trustworthiness” [42]. To this end, *investigator triangulation* was employed by involving all authors of the study in the coding and analysis processes. In addition, *data triangulation* was implemented by conducting interviews not only with bereaved children but also with their surviving parents. Another strategy used in this study was *prolonged engagement*, through the conduct of in-depth interviews lasting between 40 and 120 min. The extended duration was intended to foster trust and openness between the interviewer and interviewee, thereby enabling the emergence of rich, nuanced data. Finally, in line with the principle of *reflexivity*, we maintained a field journal throughout the interview period, in which we engaged in critical self-reflection and documented feelings, emotions, and thoughts before, during, and after the interviews (e.g., the first author’s experience as a mother; the second author’s sociocultural background—belonging to a minority group, and its potential impact; the third author’s professional experience working with children). This was conducted in order to examine whether and how these might influence the interpretations of the findings.

### 2.7. Findings

The analysis indicated that patterns of support from peers toward bereaved children lay on a continuum ranging from support, through an unintentional lack of support, to a deliberate lack of support (bullying).

1. **Support Visits and Expressions of Condolences in the Initial Phase**

In most interviews, the *shiva* (seven-day mourning period) was the first point children referred to in relation to the social dimension. In most cases, classmates and peers from the same grade came to the *shiva*, some accompanied by school staff and/or parents. The presence of classmates was experienced as an expression of support in a difficult time and was sometimes even perceived as a pleasant and highly appreciated surprise. Rafael described it as follows:

I have to say, I found out a few days later… that they had organized a delegation of kids. There was a list of kids who wanted to volunteer to come to my *shiva* after school. Plus teachers. It was crazy… One of my biggest memories is one evening when I was sitting with a girl from my class and then my first-grade homeroom teacher came. And for context—she had left the school because of COVID. She was quite old and hadn’t been at school since. It was pretty cool… it was like, oh my God!! (Rafael, 12, lost his father to cancer at 10)

It appears that peer support during the *shiva* created a kind of protective envelope against loneliness for the children. Rotem described it as follows:

It was really fun because throughout the *shiva*, the whole *shiva*, they came… like, the whole class came in groups to my house… all the kids from the class came and even kids from the parallel class… they were really sweet… and we had a really good *shiva* thanks to them… loads of people came, his friends, her friends… really a lot of people. And it was fun; it wasn’t a traumatic event. (Rotem, 14, lost her father to cancer at age 12)

Thus, at the initial stage of the *shiva*, classmates and school staff seemed to know how to respond and be present in a supportive way. With the end of the *shiva* and the return to school, however, bereaved children began to experience ambivalence about re-encountering their classmates. Rotem, for example, described it in the following way:

It was great, I think, to go back to school. Very hard. But it was good for me because, like… I had this very supportive class. And we were all there together, like a group of friends, and it was really fun… and they really supported me.

Rotem’s account reflects an ambivalent experience regarding the return to school, as she simultaneously uses the words “hard” and “great.” Nonetheless, alongside the natural difficulty of returning to school after the *shiva*, her friends served as a pillar of support she could lean on. Similarly, Lior, age 16, who lost her father to the COVID-19 pandemic, emphasized the importance of friends’ support when asked what helped her in returning to school after the *shiva*: “Mainly, meeting friends helped, and just in general, having a routine suddenly… that I go back there every day…”

In practice, support from friends formed an integral part of the process of returning to everyday routine. At the same time, participants described a decline in school-based support as more time passed since the loss. As Dganit, mother of Netanel, age 9, who lost his father to cancer, explained:

The worst thing about bereavement is time. Because as time goes by, there’s this sort of feeling in the public that time has passed and we’re fine now. Time does *not* heal! There’s this stupid saying that “time heals” or “it’s only a matter of time”… complete nonsense. I think the emphasis shouldn’t be only on the first year. On the contrary. I mean also in the first year, but also afterward—when everyone forgets, when the credit runs out. A bereaved child is a bereaved child in the second year and the third and the fourth and the tenth. (Dganit, mother of Netanel, age 9, who lost his father to cancer)

Thus, in most cases, the initial response was experienced as appropriate, yet the gradual decrease in support over time was described as a significant challenge. In addition, some participants reported more complex responses, as will be described below.

2. **Unintentional Lack of Support**

Alongside the receipt of support, some children, especially those in the younger grades of elementary school, described complex feelings in their encounters with peers that appeared to stem from a lack of understanding about how to respond appropriately. For example, Oriya, age 8.5, who lost his father to cancer and returned to school during the *shiva*, described: “My friends… were really like, “Is it true that your dad died?” like, “What, what, what?” and they really didn’t know how to react…”

Similarly, Yarden and Alma, two siblings aged 10 and 13.5 who lost their father to sudden cardiac arrest, described feelings of helplessness in the face of their classmates’ reactions at school, and the difficulty of dealing with peers’ expectations that they would behave in the way “expected” of someone who is grieving—that is, to be sad:

My friends just don’t know how to comfort, okay? They started telling me about their great-great-grandfather who died… I understood they were trying to help me, but it didn’t help. Why are you telling me… more stories?! Like, when… I’m in the middle of this now… Losing a grandparent is also a big sorrow, but… I just felt that… I just felt they weren’t helping. That’s it (smiles shyly). It just made me sadder to hear about it. (Yarden, 10, lost his father to sudden cardiac arrest at the age of 7)

After class, two boys came up to me and said something like: “Why are you happy if your dad died?” And I answered: “Because it’s my birthday and I want to be happy on my birthday.” And they just walked away. (Alma, 13.5, lost her father to sudden cardiac arrest at the age of 10)

These accounts suggest that the social environment may hold certain expectations of the bereaved child, while the child themself may simply wish to return to normalcy. Peers do not know how to offer comfort, and the result is a sense of loneliness and being misunderstood. Another example concerns peers’ use of dark humor, which the bereaved child experiences as lacking empathy. Dori, who lost her mother to cancer at age 17, described the atmosphere at school as follows:

I expected that people… wouldn’t talk about the illness, that people would be a bit more sensitive, but it wasn’t something I could really expect… because they made jokes about the illness. There’s something people aren’t aware of enough, in my opinion… that cancer is categorized as a kind of joke. And it’s not something I encountered only once, like only at school, but also when I moved to the army, and I found myself… every time there were those kinds of jokes, it was as if it created a hole in my heart… And then I thought: they’re not sensitive enough. These are people I probably shouldn’t rely on … (Dori, lost her mother to cancer at age 17)

Dori describes how insensitive reactions and dark humor made it difficult for her to trust others and reduced her willingness to share her pain. It is reasonable to assume that one possible consequence is an experience of distance between her and her peer group. Interestingly, when dark humor was used by others, it was often experienced as hurtful; however, when the bereaved child themself initiated the use of dark humor, it sometimes served as a helpful coping strategy. At the same time, some children described how their own use of dark humor posed a challenge for their non-bereaved peers:

I like making dark jokes about my dad (giggles). It’s not nice but… my friends know it comforts me… so they go along with it, they’re like… they get it and, like, yeah. At first, they felt awkward about it, but now they’re okay with it. (Alma, 13.5, lost her father to sudden cardiac arrest)

Alma’s words indicate that, although other children initially recoil from dark humor, they eventually allow it as part of their desire to support and be present with their bereaved friend. Another example comes from Shani, mother of Shoval, age 8.5, who lost his father to cancer at the age of one and a half. Shani describes his use of dark humor with his classmates, while wondering to what extent they actually understand it:

(In response to a question about coping at school): More as dark humor, I think. Not in a serious way. There was some school trip or something… someone said, “My dad isn’t coming to the trip,” so he said, “My dad isn’t coming either; my dad is in the cemetery.” His own jokes that probably only he understands. (Shani, mother of Shoval, age 8.5, who lost his father to cancer at the age of 1.5)

Dark humor thus appears to function as a coping strategy, yet peers’ responses to it are varied. They range from understanding and support to misunderstanding and the inappropriate use of dark humor. It seems that the question of *who* initiates the dark humor is a key factor.

3. **Deliberate Lack of Support—Bullying**

Alongside accounts of support, awkwardness, and helplessness, two participants also described explicit experiences of teasing that deliberately targeted their bereavement. For example, Netanel, age 9.5, who lost his father to cancer, recounted:

He told me… that I shouldn’t talk because I don’t even have a dad… We had a fight (with a boy from my class) and afterwards he said to me, “Uh… shut up, because your dad isn’t even alive. (Netanel, 9.5, lost his father to cancer at the age of 7.5)

These instances of teasing occurred mainly in situations of struggles and conflicts, in which peers used their knowledge of the child’s orphanhood as a weapon to hurt them during disputes. Yarden described a similar experience:

We argued, like really… like I made her mad about something. She’s a girl who was in my class. And suddenly she said to me… like we were really fighting because something I said to her really annoyed her. I don’t even remember what it was. And then she said to me, “At least I have a dad,” and that made me sad… It wasn’t okay… I just didn’t know what to say. (Yarden, 10, lost his father to sudden cardiac arrest)

These accounts suggest that, in the heat of ordinary school conflicts, peers sometimes draw on their knowledge of the parental loss and turn this point of vulnerability and pain into a means of “silencing” the bereaved child. Confronted with such remarks, the bereaved child is often left speechless and struggles to respond.

In sum, the findings reveal three patterns of peer response in the school context. First, support, expressed through coming to the *shiva* and offering condolences and comfort around the period of return to school. Second, a lack of understanding among peers about how to offer comfort, as well as limited understanding of dark humor as a coping strategy used by bereaved children that led to unintentional lack of support. Third, teasing and bullying directed at the bereaved child by peers that eventually resulted in an intentional lack of support.

### 2.8. Linguistic Analysis as an Extension and Validation of the Grounded Theory Analysis

As noted, in addition to grounded theory analysis, a linguistic analysis was conducted. Its findings supported and extended the grounded theory analysis, as detailed below.

The analysis of semantic fields featuring collocations with *teacher* and *teachers.* The strongest and most salient collocations that the conscientious participants used with the lexemes *teacher* and *teachers* revealed four semantic fields: Communication and support, miscommunication, empathy and sensitivity, and teacher professional development. In addition, the adverbial intensification was used to strengthen the ideas of the importance of teacher professional development in addressing students’ grief and loss, and of the school’s inability to provide the same level of support and empathy for students who experienced a parent’s death: “And at my school there wasn’t really a support system despite [the teacher’s] efforts;” *Either the teachers will say it alone, or he will say it together with his teachers*”; “*This is something I really missed, because I came and didn’t know what they told the children in my class and what they didn’t. I felt very uncertain*”. As seen, the linguistic findings supported and extended the grounded theory findings.

## 3. Discussion

This study examined the experience of social support among parentally bereaved children in elementary and middle school, using dyadic parent–child interviews. The findings indicate that the presence or absence of social support plays a significant role in shaping children’s overall experience, both in the initial period following the *shiva* and over the longer term. The main contribution of this study lies in conceptualizing the axis of social support as a continuum ranging from support to lack of support from peers toward bereaved children—whether unintentional or deliberate. The findings are discussed in light of the Dual Process Model of Coping with Bereavement [13,14] as well as Rubin’s Two-Track Model of Bereavement [15,16].

### 3.1. Patterns of Social Support from Peers Toward Bereaved Children

**Figure 1:**
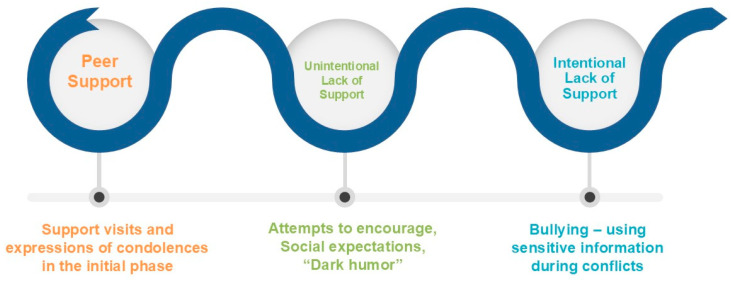
Social support patterns of school peers toward children who have lost a parent.

As noted, the analysis of peer support at school revealed three patterns of response among classmates: support; an unintentional lack of support, reflecting a lack of understanding about how to offer comfort; and a deliberate lack of support, expressed through teasing. Figure 1 presents the “Peer Support Continuum,” illustrating patterns of peer responses toward bereaved children. It enables a more precise orientation when examining how peers respond to a child coping with the loss of a parent.

The findings of this study resonate with previous research. For example, Lytje and Dyregrov [43] highlight the relief that supportive social routines at school can offer, functioning as a source of support, distraction, and containment for bereaved children. The present findings are also consistent with prior work documenting a shared sentiment among many participants that their loss differentiates them from their peers, both because they are often the only ones in their social environment who have lost someone close [26], and because of the loneliness that bereavement brings with it [27]. Likewise, the current study aligns with previous findings indicating that orphaned children may be subjected to deliberate teasing specifically around the topic of their bereavement [30].

One way to frame these findings is through the relational lens proposed by Ribbens McCarthy, Woodthorpe, and Almack [44], who argue that the impact of death should be understood not only as an individual psychological process but as something that continues to be negotiated within ongoing relationships and social contexts. From this perspective, the bereaved child’s experience at school is not merely an internal adjustment to parental loss, but part of a wider “aftermath of death” that unfolds in everyday interactions with peers and adults. The peer responses identified in this study—ranging from supportive presence, through misunderstandings and awkward attempts at comfort, to deliberate teasing—can thus be viewed as relational practices that shape how bereavement is lived, recognized, or delegitimized in the school environment. In line with Ribbens McCarthy et al.’s call to extend bereavement paradigms beyond the individual [44], our findings highlight that children’s grief is co-constructed within classroom and school microsystems, where the extent to which their loss is acknowledged, normalized, or weaponized by others has profound implications for their sense of belonging, voice, and ongoing relationship with the deceased parent.

These findings resonate with Morse’s analysis of “grievability [45],” which highlights how social and institutional contexts shape whose loss is publicly acknowledged and emotionally validated. In the school context, peers’ and teachers’ responses can be understood as part of this broader politics of recognition, whereby some children’s grief is supported while other aspects of their bereavement remain invisible or even mocked.

When interpreted in light of the Dual Process Model of Coping with Bereavement [13,14], the patterns of peer support identified in this study appear to intersect with both orientations posited by the model. When social responses are supportive, they tend to promote restoration-oriented coping. However, when children experience a lack of support, especially deliberate nonsupport involving teasing, this may “force” them back into a loss-oriented focus. For instance, when, in the midst of a conflict, a peer invokes the bereaved child’s parental loss (e.g., by saying that the child has no father) as a way to “win” a struggle, the bereaved child is involuntarily thrust back into a focus on the loss and the relationship with the deceased. This occurs despite the child’s wish, in the school context, to concentrate on restoration and everyday life.

In other words, conflicts with peers can act as triggers that re-activate the experience of loss through peers’ responses. Whereas the Dual Process Model conceptualizes the bereaved individual as oscillating between loss-oriented and restoration-oriented coping, the present findings suggest that, in the school context, an orphaned child may be pushed back into loss-oriented processing against their will as a direct result of peers’ reactions. In this sense, the child’s sense of control over these shifts is undermined and may generate particularly high levels of distress. This underscores the crucial role of school staff in promoting optimal support for bereaved students.

Furthermore, findings may be interpreted in light of the Two-Track Model of Bereavement [15,16] that serves as a conceptual, theoretical, research, and clinical framework for understanding adaptation to loss. According to which, there are two parallel tracks of coping: one externally oriented, focusing on the bereaved individual’s bio-psycho-social functioning, and one internally oriented, centering on the relationship with the deceased [16]. Viewing the “Peer Support Continuum” through the lens of Rubin’s dual-track paradigm underscores its relevance to the first, functioning-focused track. As the findings suggest, peers’ response patterns constitute a significant component influencing bereaved children’s mourning processes—during the *shiva*, upon returning to school, and over the longer term. The results indicate that peer responses shape the experience of returning to school routines, the extent and nature of bereaved children’s re-engagement in social life, their sense of being understood, and their ability to express themselves, as reported by participants. Thus, when a bereaved child experiences support from peers, they may return to functioning more quickly and adaptively. In contrast, an absence of support, whether intentional or unintentional, can hinder reintegration into the school environment and at times contribute to feelings of isolation or difficulty resuming academic activities. Accordingly, the level of support or lack of support from peers appears to influence the quality and nature of the child’s return to functioning, corresponding to the first track in Rubin’s paradigm. Again, this underscores the crucial role of school staff in mediating and fostering social learning processes that promote optimal support for bereaved students, as elaborated below.

### 3.2. The Importance of Training School Staff

In light of the study’s findings, the importance of training school staff to mediate the relationship between a bereaved child and their classmates emerges clearly—an implication that echoes previous research [5,46]. Prior studies have shown that school staff often struggle to address the social, academic, and emotional consequences of bereavement for children [47,48,49]. One reason for this difficulty is that support efforts tend to be uncoordinated and that staff lack appropriate, tailored training [50]. An examination of teachers’ coping with bereaved children in school during the COVID-19 pandemic identified three primary arenas of coping: an intrapersonal arena (the teacher in relation to themself), an interpersonal arena (the teacher in relation to the bereaved student), and a group arena (the teacher in relation to the class). In each of these arenas, teachers were found to employ, simultaneously, both emotion-focused and problem-focused coping strategies [37].

Furthermore, bereaved children reported that over time, the level of support they received from both teachers and peers declined. As time passed, the school community and classmates appeared to “move on” and, in effect, forget the event, while the children themselves continued to grapple with their bereavement and its consequences [32]. To enable teachers to provide guidance and direction to peers on how to support a bereaved child, they must be equipped with both theoretical and practical knowledge [51]. A deeper understanding of the roles and responsibilities of different professionals within the school setting can facilitate the development of a structured program aimed at improving support for bereaved children. Author proposed a model in which teachers serve as the primary school-based agents responsible for the bereaved student’s well-being, while school-based mental health professionals (e.g., counseling staff, psychologists) provide teachers with practical and emotional support [5]. In cases of complicated grief, however, mental health professionals would assume the primary therapeutic role, with teachers positioned as secondary supporters [5].

It appears that the development of clear, research-informed policies is essential for optimizing support for bereaved children in schools. In this context, the “Peer Support Continuum” proposed in the present study can serve as a valuable conceptual and practical tool for school staff. It can help them identify and assess the type of peer response that a bereaved child is receiving in their school and respond appropriately and promptly. Thus, instances of lack of support stemming from misunderstanding may call for guidance and facilitated dialog between staff and students, whereas deliberate nonsupport and teasing require a more assertive response to safeguard the bereaved child’s well-being.

### 3.3. Limitations

Several limitations of the present study should be noted. First, the study population consisted of participants who received support from the Sunflowers Organization. It is possible that their experiences differ from those of bereaved children who do not receive emotional support in a similar framework. At the same time, qualitative research does not seek to uncover an objective truth, but rather to understand and construct the meanings and interpretations of participants. We believe that the study has the potential for transferability (which replaces the principle of generalizability in positivist research paradigms), given the diversity of participants in terms of age and the triangulation carried out between parents and children. Second, the study focused on the narratives of children in elementary and middle school. Future research could examine this issue among additional age groups, such as high school students, as well as across other sectors and also by examining gender differences. Because developmental processes in childhood and adolescence are strongly expressed in the social domain, different ages are associated with different types of social challenges. Thus, future studies may further contribute to understanding the social experience of children who have been orphaned by the death of one or both parents at ages not included in the present study. Third, the interviews were conducted in the presence of a parent, which may have affected the children’s openness in talking about their experiences. However, it is important to note that both children and parents were given the opportunity to add information privately (upon parent’s consent), and that ethical considerations were decisive in resolving this dilemma.

### 3.4. Importance of the Study

Given the increasing prevalence of orphaned children worldwide [52] and in Israel, there is a substantial need to develop evidence-based knowledge to support them across different life domains, including the school setting. This study deepened the understanding of the experiences of children who are orphaned by the death of a parent, and, in particular, how they perceive the social support provided by their peers. The findings highlight the complex social reality bereaved children must navigate upon returning to school, spanning a continuum from support to intentional lack of support in the form of teasing. This underscores the crucial role of the educational staff in mediating relationships between bereaved children and their peers.

An additional contribution of this study lies in distinguishing between a lack of social support that stems from children’s misunderstanding of how to provide support and deliberate intentions to harm bereaved peers. This distinction allows for differentiated responses at the level of school policy and practice. Thus, children who act out of misunderstanding require mediation and guidance, whereas those who intentionally cause harm require a more firm and corrective response so that such behavior is not tolerated. The study further contributes to guiding parents, educational staff, and mental health professionals in anticipating patterns of relationships that may emerge following parental loss within the school and family context. In this sense, the study provides an opportunity for parents as well to better understand this dimension of their children’s lives. Overall, the findings illuminate the need to address the current lack of clear policy regarding how schools and educational staff should respond to and support orphaned students. It would be appropriate to promote the development of a clear, research-informed policy and practical protocol to guide school staff in their work with bereaved children and their families. Such a framework could help ensure that support for parentally bereaved children is structured, evidence-based, and optimally responsive to their needs.

## Figures and Tables

**Table 1:** Participants’ Demographic Characteristics.

Pseudonym	Age at Interview (Years)	Age at Loss	Time Since Death	Gender	Deceased Parent	Circumstances of Death	Parent Age (Years)	Education Level
Rotem	14	12	2	Female	Father	illness	56	High school
Dori	18	17	1	Female	Mother	illness	58	Academic
Ofir	12	6.5	5.5	Male	Mother	illness	47	Academic
Shoham	14	12	2	Female	Mother	Sudden	49	Academic
Naomi	8.5	6.5	2	Female	Mother	Sudden	49	Academic
Shoval	8.5	1.5	7	Male	Father	illness	40	Academic
Rafael	12	10	2	Male	Father	illness	49	Academic
Oriya	8.5	6.5	2	Male	Father	illness	49	Academic
Lior	16	12	4	Female	Father	illness	53	Academic
Gefen	9	8	1	Female	Father	illness	40	Academic
Tal	11	9.5	1.5	Female	Father	illness	46	Academic
Adi	11	9.5	1.5	Female	Father	illness	46	Academic
Alma	13.5	10	3.5	Female	Father	Sudden	49	Academic
Yarden	10	7	3	Male	Father	Sudden	49	Academic
Netanel	9.5	7.5	2	Male	Father	illness	43	Academic
Alona	16	13	3	Female	Father	Sudden	48	High school
Alon	9	1	8	Male	Mother	Illness	40	Academic
Kfir	7.5	6.5	1	Male	Father	Sudden	50	High school
Maayan	10	6	4	Male	Father	Sudden	40	Academic
Carmi	13	12	1	Female	Father	Sudden	49	Academic

**Table 2:** Semantic field analysis: coding method, subcategories, and examples.

Distinctive Linguistic Markers	Subcategory	Coding Method	Examples
**Semantic fields to evaluate the participants’ view of the teacher’s role in the program**	Semantic Field of *Communication and Support*	The lexemes that collocate with *teacher* and *teachers* to denote child-teacher communication	Told, informed, sat (with me), supported, was (with me), together, the best, etc.
	Semantic Field of *Miscommunication*	The lexemes that collocate with *teacher* and *teachers* to denote miscommunication	Did not know, asks, alone, etc.
	Semantic Field of *empathy and sensitivity*	The lexemes that collocate with *teacher* and *teachers* to denote empathy and sensitivity	Special attitude, explain, understand, etc.
	Semantic Field of *guiding teachers*	The lexemes that collocate with *teacher* and *teachers* to denote *teacher professional development*	More to do, professional, guidance, change, etc.

## Data Availability

The data presented in this study are available on request from the corresponding author. (the data are not publicly available due to privacy restrictions).
